# Retinal and Choriocapillary Vascular Changes in Early Stages of Multiple Sclerosis: A Prospective Study

**DOI:** 10.3390/jcm10245756

**Published:** 2021-12-09

**Authors:** Daniela Montorio, Roberta Lanzillo, Antonio Carotenuto, Maria Petracca, Marcello Moccia, Chiara Criscuolo, Antonio Luca Spiezia, Anna Lamberti, Federico Perrotta, Giuseppe Pontillo, Gilda Cennamo, Vincenzo Brescia Morra

**Affiliations:** 1 Department of Neurosciences, Reproductive and Odontostomatological Sciences, Federico II University, 80131 Naples, Italy; da.montorio@gmail.com (D.M.); robertalanzillo@libero.it (R.L.); carotenuto.antonio87@gmail.com (A.C.); maria@petraccas.it (M.P.); moccia.marcello@gmail.com (M.M.); sky569@hotmail.com (C.C.); antonapoli-94@hotmail.it (A.L.S.); anna.lamberti00@gmail.com (A.L.); feder.perrotta@studenti.unina.it (F.P.); vincenzo.bresciamorra2@unina.it (V.B.M.); 2 Department of Advanced Biomedical Sciences, Federico II University, 80131 Naples, Italy; giuseppe.pon@gmail.com; 3 Eye Clinic, Public Health Department, University of Naples Federico II, 80131 Naples, Italy

**Keywords:** OCTA, vessel density, initial demyelinating event, multiple sclerosis, prospective study

## Abstract

Optical Coherence Tomography Angiography (OCTA) abnormalities occur in multiple sclerosis (MS) over the course of the disease. OCTA investigations at early MS stages are lacking. We aimed to investigate vessel density in macular and papillary regions over two years after an initial demyelinating event (IDE). Vessel density was analyzed in superficial, deep, choriocapillaris and radial peripapillary plexus at baseline, and after one and two years. We also evaluated structural OCT parameter changes of the ganglion cell complex (GCC) and retinal nerve fiber layer (RNFL). We evaluated 30 eyes from 15 IDE patients (7 females, 8 males, mean age 28.4 ± 9.6 years) and 30 eyes from 15 healthy controls. After 2 years, we reported in the IDE group a reduced vessel density in the superficial capillary plexus, deep capillary plexus and radial peripapillary capillary plexus with respect to the baseline (coeff. β = −2.779, *p* = 0.013; coeff. β = −4.055, *p* = 0.018 and coeff. β = −2.687, *p* ≤ 0.001; respectively), while GCC and RNFL thicknesses did not change. Vessel density reduction was not associated with an expanded disability status scale (EDSS) change, relapse occurrence or magnetic resonance imaging activity. The analysis of healthy controls did not reveal any impairment in OCT and OCTA parameters over 2 years of follow-up. Retinal vascular loss occurs in patients with an IDE independently from clinical and radiological disease activity. Retinal vessel density could represent a novel early biomarker to monitor the MS pathological burden.

## 1. Introduction

Multiple sclerosis (MS) is an inflammatory, demyelinating disorder of the central nervous system with progressive neuroaxonal degeneration [1]. The availability of sensitive biomarkers to monitor pathological changes represents an unmet need in MS clinical management.

The retina, showing similar anatomical and physiological features as the brain, reflects cerebral changes occurring in MS patients [2,3]. The use of optical coherence tomography (OCT) in MS studies facilitates the detection, in a non-invasive and rapid way, of the changes to neuroretinal structures, such as ganglion cell complexes (GCC) and retinal nerve fiber layers (RNFL), at different stages of this disease [4].

The OCT parameters allow investigation of MS pathophysiological mechanisms, both at early disease stages with prominent inflammatory activity and later on, as neurodegeneration advances [5]. RNFL and GCC also allow for stratifying the patients for different treatment choices [6,7]. Several reports and longitudinal evaluations demonstrated a significant retinal neurodegeneration, confirmed by thinning of GCC and RNFL [8,9].

Previous studies using magnetic resonance imaging (MRI) suggested cerebral hypoperfusion as a possible contributor to the MS disease course [10,11]. Increasing evidence outlines the common morphological and physiological characteristics of retinal and cerebral vascularization, thus, demonstratingthat changes in retinal vascularization could be a potential marker of the cerebrovascular state [12,13]. Therefore, retinal perfusion assessment may provide insight into cerebral hypoperfusion, possibly contributing to further unraveling MS pathogenesis and disease severity.

OCT angiography (OCTA), a non-invasive imaging technique, is a useful tool to detect and to quantify the retinal and choriocapillary blood flow in macular and papillary regions in neurodegenerative diseases [13]. Previous studies demonstrated a significant involvement of retinal and choriocapillaris vascularization that correlated with disease disability in MS patients [14,15]. Until now, few studies analyzed retinal and choriocapillaris vascular networks in clinically isolated syndromes through pooled analysis with relapsing remitting MS, but no study focused on their vascular density changes in longitudinal settings. A previous report highlighted the role of impaired retinal perfusion as a possible biomarker in monitoring the pathological changes in MS [16].

In line with these data, we previously showed a significant vessel density (VD) reduction of the radial peripapillary capillary plexus in patients with an initial demyelinating event (IDE) in respect to controls [17]. Building on this finding, we hypothesized that the observed retinal VD reduction, already present at the IDE, would further worsen over time.

To explore this hypothesis, we longitudinally investigated retinal and choriocapillary VD of macular and papillary regions in patients with an IDE by means of OCTA. This study would better define the vascular involvement in MS pathogenesis and may eventually unveil a possible biomarker of vascular pathological changes.

## 2. Materials and Methods

### 2.1. Subjects

This is a pilot prospective longitudinal study over 2 years. We enrolled patients with an IDE at the “Federico II” University of Naples MS Centre, from January to December 2018. An IDE was considered to be the first neurologic symptom referable to demyelination in the central nervous system, lasting for at least 48 hours, regardless of whether patients met relapsing–remitting MS or a clinically isolated syndrome diagnosis following the 2017 McDonald criteria.

Each patient underwent a complete neurological and ophthalmological examination at baseline, and after 1 year and 2 years from an IDE, including the assessment of physical disability through the expanded disability status scale (EDSS), evaluation of best-corrected visual acuity, slit-lamp biomicroscopy, fundus examination and spectral domain OCT (SD-OCT) and OCTA. Ophthalmological evaluation was blinded to subjects’ clinical status.

We collected MRI data from the radiological database of our institution, considering exams that were performed yearly for the standard of care disease monitoring. Radiological activity was defined as presence of gadolinium enhancing lesions and/or presence of new, or enlarging, T2 lesions in the follow-up scans in comparison with the baseline MRI.

We excluded patients with a history of optic neuritis, glaucomatous optic neuropathy and patients with signs of subclinical optic neuritis based on inter-eye difference in order to avoid a bias related to optic nerve direct damage [18,19,20].

Patients with a relapse and/or corticosteroid use in the previous month were excluded. Other exclusion criteria were: the presence of systemic vascular diseases (high blood pressure, diabetes and heart disease), clinically relevant lens opacities, low-quality images obtained with SD-OCT and OCTA, myopia greater than 6 diopters, history of intraocular surgeries, vitreo-retinal and retinal vascular diseases, uveitis, and congenital eye disorders.

Lastly, we enrolled each age and sex-matched 15 healthy controls that presented normal neurological (including MRI) and ophthalmic examinations. The healthy controls were obtained from a large database at Eye Clinic from previous research projects. They underwent brain MRI scans at the neurology department. Moreover, OCT and OCTA evaluations were performed at baseline, after 1 year and 2 years. Two independent observers (GC; DM) carefully reviewed the SD-OCT and OCTA images.

The study was approved by the Institutional Review Board of the University of Naples “Federico II” and all investigations adhered to the tenets of the Declaration of Helsinki (protocol number: 142/19). Written informed consent was obtained from each of the subjects enrolled in the study.

### 2.2. Optical Coherence Tomography

RNFL and GCC thicknesses were obtained with SD-OCT (software RTVue XR version 2018.1.1.60, Optovue Inc., Fremont, CA, USA). The circumpapillary RNFL was analyzed by the optic nerve head map protocol using a 3.45 mm radius ring centered on the optic disc. The GCC thickness was analyzed from the internal limiting membrane to the outer boundary of the inner plexiform layer, the scan was centered 1-mm temporal to the fovea and covered a 7 × 7 mm^2^ area over the macular region [21].

Each OCT scan was evaluated according to APOSTEL recommendations and the OSCAR-IB protocol for quality control [22,23]. These guidelines were adapted for our device.

### 2.3. Optical Coherence Tomography Angiography

OCTA images were performed by the Optovue Angiovue System (software ReVue XR version 2018.1.1.60, Optovue Inc., Fremont, CA, USA) that is based on a split-spectrum amplitude de-correlation algorithm which uses blood flow for intrinsic contrast [24].

The macular capillary network was evaluated from a 6 × 6 mm^2^ scan centered on the fovea and the AngioAnalytics™ software automatically calculated the VD that represents the percentage area occupied by the vessels in the analyzed region [25].

The OCTA software analyzed the whole area of the macular region in each vascular network of the retina, namely the superficial and deep capillary plexuses, and choriocapillaris. The Angio-Vue disc mode automatically calculated the VD of the radial peripapillary capillary plexus analyzing the whole papillary region with a scanning area of 4.5 × 4.5 mm^2^ centered on the optic disc (whole image).

VD for the radial peripapillary capillary plexus was analyzed in the superficial retinal layers and extended from the inner layer membrane to the retinal nerve fiber layer posterior boundary [26].

The OCTA device included the 3D Projection Artifact Removal algorithm to remove projection artifacts, for improving depth resolution on OCTA signal and then distinguishing vascular plexus-specific features. OCTA images with a Signal Strength Index less than 80 and residual motion artifacts were excluded from the analysis

### 2.4. Statistical Analysis

Statistical analysis was performed with the Statistical Package for Social Sciences (Version 25 for Windows; SPSS Inc, Chicago, Ill, USA). Clinical and MRI features for included subjects were presented as means, medians or proportions, as appropriate. Normality distribution was assessed through a Shapiro–Wilk test and model residuals were visually inspected to ensure model homoscedasticity.

We explored VD changes over time in each retinal vascular network (superficial capillary plexus, deep capillary plexus and radial peripapillary capillary plexus) and in choriocapillaris, as well as structural OCT parameters (GCC average and RNFL average) changes through general linear models, including age, sex, EDSS at baseline, occurrence of MRI activity or relapse over the follow-up as covariates and time points as factors of interest to evaluate.

General linear models were used to evaluate VD changes of retinal and choriocapillary vascular networks in healthy controls over two years of follow up. Correlations between OCTA parameters and MRI activity, relapse occurrence and EDSS were assessed using linear mixed models. The subject was included in all models as a random factor to account for within-subject inter-eye correlation. The agreement between two observers in the measurement of SD-OCT and OCTA parameters was assessed using the intraclass correlation coefficient. A *p* value < 0.05 was considered statistically significant.

## 3. Results

### 3.1. Baseline Features

Thirty eyes from 15 patients with an IDE (7 females, 8 males; mean age 28.4 ± 9.6 years) and 15 healthy controls (8 females, 7 males; mean age 27.2 ± 8.7 years) were analyzed. No significant difference in terms of age and sex was found between the two groups. Demographic, clinical features and OCTA findings at baseline are summarized in Table 1, Table 2 and Table 3. The VD in the radial peripapillary capillary plexus was significantly lower in those patients with an IDE compared with the healthy controls (coeff. β = −3.102; *p* = 0.003).

### 3.2. Follow-Up Features and Association between Clinical/Radiological Activity and OCT/OCTA Changes

The analysis of the healthy controls revealed no significant change over two years in both the OCT and OCTA parameters (Table 2). The intraclass correlation coefficient for both SD-OCT and OCTA parameters was 0.92.

In patients with an IDE, best-corrected visual acuity after 1 and 2 years did not differ from the baseline (*p* = 0.10). Similarly, GCC and RNFL thicknesses did not change over time (Table 3). Moreover, no ocular or metabolic diseases related to ocular side effects developed over the follow-up.

Only one patient experienced a relapse 10 months after the baseline OCT examination and received pulsed steroid therapy. Thirteen of the 15 patients underwent brain MRI scans for disease monitoring over the study period, with one patient showing a gadolinium enhancing lesion between the baseline and year 1, and a second patient presenting four new T2 lesions between year 1 and year 2.

**Figure 1:**
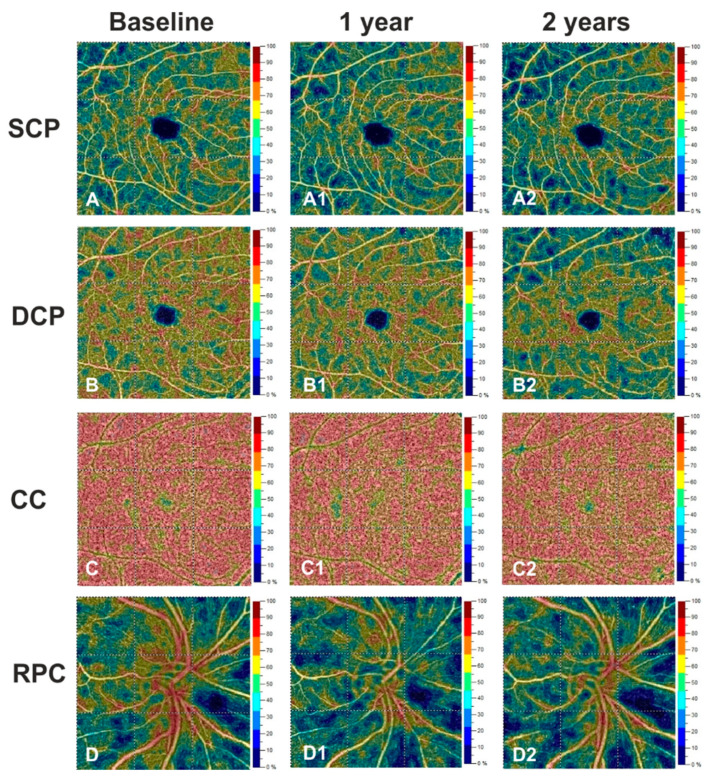
Right eye of a case patient with IDE (27 years-old male) shows a marked vessel density reduction for superficial capillary plexus (SCP) (**A2**), deep capillary plexus (DCP) (**B2**) and radial peripapillary capillary plexus (RPC) (**D2**) at years 2 respect to baseline (**A**,**B**,**D**) and year 1 (**A1**,**B1**,**D1**). VD in the choriocapillaris (CC) did not change over the follow-up (**C**,**C1**,**C2**).

While we did not show any change in OCTA measures in the healthy controls, in patients with an IDE we identified a decrease in VD in the superficial, deep capillary plexuses and radial peripapillary capillary plexus (coeff. β = −2.779; *p* = 0.013, coeff. β = −4.055; *p* = 0.018 and coeff. β = −2.687; *p* < 0.001, respectively) after 2 years, compared with the baseline. These changes were confirmed when comparing measures obtained after 2 years with those observed at 1 year after the baseline (coeff. β = −2.543; *p* = 0.017, coeff. β = −3.861; *p* = 0.015 and coeff. β = −1.853; *p* = 0.005, respectively) (Figure 1, Table 3). Conversely, VD of the choriocapillaris did not show significant differences from the baseline at any time point. EDSS, MRI activity and relapse occurrence did not correlate with OCTA and OCT changes.

The agreement between the two observers for measuring the SD-OCT and OCTA parameters was excellent, with an intraclass correlation coefficient of 0.92.

## 4. Discussion

To our knowledge, this is the first longitudinal study to investigate the retinal and choriocapillaris VD by means of OCTA in patients with an IDE.

We reported no changes for structural OCT measures over 2 years after an IDE. This finding was not surprising as it confirms previous results revealing the absence of abnormalities in retinal neuronal components in patients with an IDE respect to the controls [17]. Conversely, several reports analyzing different MS phenotypes reported a significant annualized rate change for GCC and RNFL over time, suggesting a possible retrograde trans-synaptic degeneration as the mechanism of retinal neuronal damage [27,28,29,30].

On the other hand, when exploring OCTA changes over time, we reported a progressive VD reduction in almost all retinal areas over the second year following an IDE. These findings would suggest a vascular involvement beyond neurodegeneration and neuroinflammation in an IDE.

Indeed, previous studies have already outlined, using arterial spin labeling MRI scans, the contribution of vascular factors in the detection of other neurodegenerative disorders, such as Alzheimer’s disease, mild cognitive impairment, and Parkinson’s disease [31,32]. Moreover, several studies reported the close relationship between the alterations in retinal perfusion by OCTA and the systemic vascular impairment involving cardiovascular and central nervous systems [33,34]. These results supported the hypothesis that retinal VD could represent a valid biomarker to detect the early systemic vascular damages.

Perfusional changes and neurodegeneration are intermingled processes in MS and disentangling their dynamic longitudinal changes is challenging.

Although the pathogenic mechanisms are still a source of debate, our preliminary results showed the possible involvement of retinal vascular impairment in an IDE, but this would not exclude the possible retinal structural alterations that could already be present but still in the subclinical phase. These findings would support the importance of structural and vascular parameters as complementary information.

Our results did not show any significant change in choriocapillaris in an IDE over time, excluding a possible involvement of this vascular network in the MS disease course. Similarly, Feutch et al. did not identify any significant differences in the VD of choriocapillaris among the healthy controls, eyes with and without optic neuritis, but demonstrated in MS patients a positive correlation between higher choriocapillaris VD and the occurrence of relapse [16]. In our study, we only reported a clinical relapse in one patient, hence, we may not provide firm conclusions about the association between relapse and VD abnormalities.

The evaluation of healthy controls in this study contributed to confirm the relevance of our results. The absence of any change in both OCT and OCTA parameters in the healthy controls demonstrated that longitudinal OCTA alterations occurring in the IDE group are closely related with the demyelinating event and ensure that longitudinal changes observed in the IDE group may not relate to systematic biases eventually introduced by the OCTA software.

We do acknowledge that this study is not without limitations. Firstly, the small sample may have reduced the sensitivity in detecting OCT abnormalities in MS patients at very early stages. Secondly, the presence of few IDE cases with MRI activity and relapse occurrence also limited our results to a specific MS subgroup. Moreover, the IDE patients underwent therapy during follow-up. This may have determined a possible effect on the vascular density.

## 5. Conclusions

The results of this study showed in IDE patients a retinalblood flowrarefaction that maybe could reflect a cerebrovascular degenerative process during the follow-up. In this scenario, the retinal VD may be an early biomarker in IDE patients.

Further longitudinal studies on larger cohorts and longer follow up periods, involving MRI activity and relapse occurrence, would be necessary to validate these results. Our future purpose will be to analyze the FAZ changes as early biomarkers in MS pathogenesis. Moreover, the correlations between OCTA and functional parameters could be a source of further study.

## Figures and Tables

**Table 1:** Demographic and clinical characteristics of patients with IDE and healthy controls.

	IDE Patients	Healthy Controls
Subjects (*n*)	15	15
Eyes (*n*)	30	30
Age, mean ± SD (years)	28.4 ± 9.6	27.2 ± 8.7
Sex (female/male)	7/8	8/7
EDSS, mean ± SD	1.81 ± 0.56	-
Onset modality		
Brainstem, N. (%)	4 (27%)	-
Pyramidal, N. (%)	4 (27%)	-
Cerebellar, N. (%)	1 (7%)	-
Sensory, N. (%)	5 (32%)	-
Bowel/Bladder, N. (%)	0 (0%)	-
Cerebral, N. (%)	1 (7%)	-
BCVA (logMAR)	0.02 ± 0.04	0.01 ± 0.03

IDE: Initial Demyelinating Event; EDSS: Expanded Disability Status Scale; BCVA: Best Corrected Visual Acuity; logMAR: logarithm of the minimum angle of resolution.

**Table 2:** Clinical characteristics of healthy controls at baseline and each time point.

	Baseline	1 Year	2 Years	Baseline vs. 1 Year		Baseline vs. 2 Years		1 Year vs. 2 Years	
				β	*p*-value	β	*p*-value	β	*p*-value
**OCT-A parameters (%)**									
*SCP Whole*	53.70 ± 3.51	52.65 ± 3.33	53.82 ± 2.88	1.043	0.177	−0.127	0.870	−1.170	0.130
*DCP Whole*	56.95 ± 3.22	57.29 ± 3.46	57.35 ± 3.98	−0.340	0.703	−0.403	0.651	−0.063	0.943
*CC Whole*	72.59 ± 3.46	73.65 ± 3.23	73.81 ± 2.69	−1.063	0.173	−1.223	0.117	−0.160	0.838
*RPC Whole*	52.54 ± 4.15	51.51 ± 4.49	52.70 ± 3.75	1.033	0.276	−0.160	0.866	−1.193	0.209
**OCT parameters (µm)**									
*GCC average*	100.73 ± 5.64	100.60 ± 5.03	101.93 ± 4.47	0.130	0.917	−1.200	0.346	−1.333	0.295
*RNFL average*	104.86 ± 5.88	104.90 ± 5.32	105.60 ± 5.03	−0.030	0.979	−0.733	0.567	−0.700	0.585

Data expressed as mean ± standard deviation. OCT-A: Optical Coherence Tomography Angiography; SCP: Superficial Capillary Plexus; DCP: Deep Capillary Plexus; CC: Choriocapillaris; RPC: Radial Peripapillary Capillary; OCT: Optical Coherence Tomography; GCC: Ganglion Cell Complex; RNFL: Retinal Nerve Fiber Layer. General linear models.

**Table 3:** Clinical characteristics of patients with IDE at baseline and each time point.

	Baseline	1 Year	2 Years	Baseline vs. 1 Year		Baseline vs. 2 Years		1 Year vs. 2 Years	
				β	*p*-value	β	*p*-value	β	*p*-value
**OCT-A parameters (%)**									
*SCP Whole*	50.23 ± 4.56	50.09 ± 3.82	47.53 ± 4.71	−0.142	0.881	−2.779	0.013 *	−2.543	0.017 *
*DCP Whole*	55.12 ± 6.07	55.02 ± 6.23	50.99 ± 6.97	−0.086	0.952	−4.055	0.018 *	−3.861	0.015 *
*CC Whole*	74.01 ± 2.20	73.37 ± 3.40	72.85 ± 4.39	−0.641	0.371	−1.226	0.121	−0.566	0.483
*RPC Whole*	49.62 ± 2.85	48.74 ± 2.24	46.90 ± 2.45	−0.877	0.144	−2.687	<0.001 *	−1.853	0.005 *
**OCT parameters (µm)**									
*GCC average*	98.23 ± 7.09	96.66 ± 10.17	94.68 ± 11.71	−1.566	0.482	−4.065	0.078	2.421	0.271
*RNFL average*	101 ± 10.44	99.53 ± 10.58	98.22 ± 8.52	−1.464	0.473	−3.345	0.154	−1.815	0.451

Data expressed as mean ± standard deviation. IDE: Initial Demyelinating Event; OCT-A: Optical Coherence Tomography Angiography; SCP: Superficial Capillary Plexus; DCP: Deep Capillary Plexus; CC: Choriocapillaris; RPC: Radial Peripapillary Capillary; OCT: Optical Coherence Tomography; GCC: Ganglion Cell Complex; RNFL: Retinal Nerve Fiber Layer. General linear models, * *p* < 0.05.
